# A Case of Spontaneous Umbilical Endometriosis

**DOI:** 10.7759/cureus.107516

**Published:** 2026-04-22

**Authors:** Jennifer J Law, Victoria C Kuritza

**Affiliations:** 1 Obstetrics and Gynecology, Loyola University Medical Center, Maywood, USA; 2 Dermatology, Medical Dermatology Associates of Chicago, Chicago, USA

**Keywords:** cutaneous, ectopic, endometriosis, umbilical, villar’s nodule

## Abstract

Umbilical endometriosis, also known as Villar’s nodule, is an atypical manifestation of endometriosis. We present a rare case of spontaneous Villar’s nodule in a young, nulliparous woman with no history of abdominal surgery or trauma who had no classic symptoms of endometriosis. Clinical examination revealed a painless umbilical nodule, and histopathologic evaluation demonstrated endometrial-type glands and stroma, confirming primary cutaneous endometriosis. Further gynecologic evaluation, including pelvic examination and transvaginal ultrasound, showed no evidence of pelvic endometriosis. We review proposed pathogenic mechanisms, discuss diagnostic considerations, and highlight the importance of prompt gynecologic referral. Early recognition with complete excision and appropriate gynecologic follow-up is essential, particularly in patients desiring future fertility.

## Introduction

Endometriosis is a disorder characterized by the presence of endometrial glands and/or stroma outside the uterine cavity and is the most common cause of chronic pelvic pain in females of reproductive age [1]. Ectopic endometrial implants can be found in many tissues but most commonly affect pelvic organs, including the ovaries, fallopian tubes, uterine ligaments, and pelvic sidewalls [1,2]. Less common is extra-pelvic endometriosis, which can occur outside the genital organs, including the lung, liver, bowel, urinary tract, diaphragm, and skin [1-5]. Cutaneous endometriosis represents less than 1% of all ectopic endometrial tissue and is further classified into primary or secondary cutaneous endometriosis [2,3]. Secondary cutaneous endometriosis is iatrogenic, where endometrial cells are seeded in scar tissue that forms at prior surgical or traumatic sites, commonly after cesarean section, hysterectomy, and laparoscopy [2,3]. Primary cutaneous endometriosis, which occurs spontaneously without preceding surgery or trauma, is far rarer, and its pathogenesis remains unknown [2,3]. Among cutaneous sites, umbilical endometriosis, also known as Villar’s nodule, accounts for 30-40% of reported cases [2,4].

Villar’s nodule is an unusual clinical presentation and is difficult to diagnose because of its rarity and nonspecific appearance [5]. Accurate recognition is important for a better understanding of its prevalence, potential etiologies, and optimal management. Here, we report a case of primary umbilical endometriosis in a patient with noncyclic bleeding and absence of classic symptoms of endometriosis (e.g., pelvic pain, dysmenorrhea, dyspareunia, dysuria, or dyschezia).

## Case presentation

A 29-year-old nulligravid woman presented to the dermatology clinic with a five-month history of a painless umbilical lesion that occasionally bled. The patient recalled two episodes of painless bleeding from the umbilical region, neither of which was associated with her menstrual cycle. She denied progressive growth of the lesion. She also denied dysmenorrhea, dyspareunia, pelvic pain, or other systemic symptoms. Her history was negative for prior surgery, abdominal trauma, and a personal or family history of malignancy.

**Figure 1:**
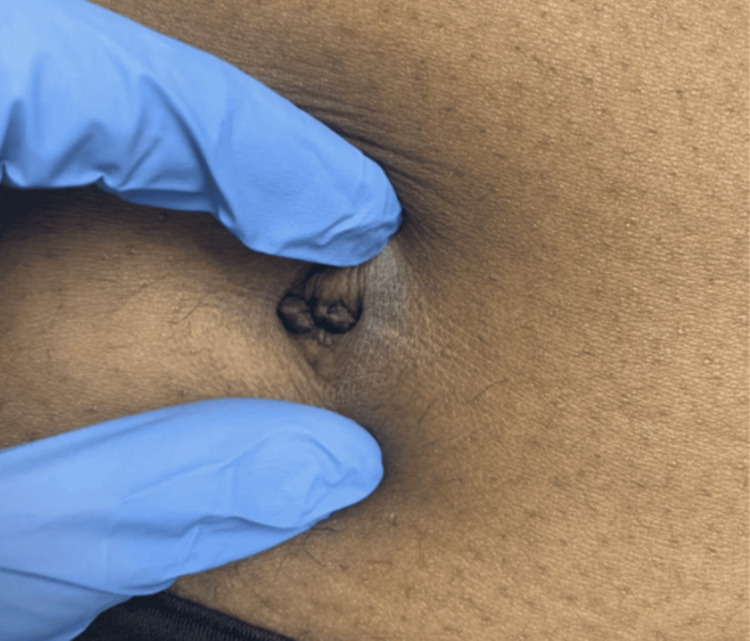
Umbilical endometriosis Macroscopic view of the umbilicus in a 29-year-old female showing an approximately 1.2 × 0.8 × 0.2 cm purple-tan exophytic lesion protruding from the umbilicus without signs of bleeding, crusting, or inflammation.

Physical examination revealed a 1.2 × 0.8 × 0.2 cm purple-tan, exophytic lesion protruding from the umbilicus (Figure 1). The initial differential diagnosis included seborrheic keratosis, skin tag, keloid, wart, and Sister Mary Joseph nodule.

The patient underwent excision of the umbilical lesion. Histopathological evaluation demonstrated isolated glands rimmed by endometrial-type stroma, consistent with primary cutaneous endometriosis. The patient underwent further workup by a gynecologist because of the diagnosis of Villar’s nodule and a family history of infertility in her mother. The workup included a normal pelvic examination with no tenderness or fullness on bimanual examination. Pelvic ultrasound also showed a normal uterus and adnexa with the absence of endometriomas. At follow-up three months later, the patient reported no recurrence of the umbilical lesion or bleeding at the umbilical site.

## Discussion

Endometriosis is histopathologically characterized by the presence of functional ectopic endometrial tissue outside the uterine cavity and can cause pain and infertility in women of reproductive age. While the exact prevalence is unknown, endometriosis is estimated to affect 0.7-8.6% of reproductive-aged women [1]. The prevalence appears to be substantially higher in women with subfertility, ranging from 9.0% to 68.0%, as well as 15.4-71.4% in women with chronic pelvic pain [1]. Clinicians should suspect endometriosis in patients with cyclic or noncyclic chronic pelvic pain, dysmenorrhea, dyspareunia, dysuria, dyschezia, or infertility associated with one or more of these symptoms [1]. Endometriosis is a chronic, often debilitating disease that shares several features with malignancy in that it is progressive, invasive, and estrogen-dependent [1,6]. Early detection and diagnosis are important, particularly in women desiring future pregnancy.

Several theories have been proposed to explain endometriosis pathogenesis. The most widely accepted is Sampson’s theory of retrograde menstruation, in which endometrial progenitor cells are shed from the uterine lining, reflux through the fallopian tubes into the peritoneal cavity, and implant on pelvic structures [6,7]. Once implanted, these cells are hormone-responsive and drive acute and chronic inflammatory reactions that can lead to pain and infertility [1,2,6]. Dridi et al. suggested that primary umbilical endometriosis may originate from ectopic endometrial glands that migrate through the peritoneal cavity via the right hemidiaphragm to the umbilicus along the falciform and round ligaments of the liver [4]. This retrograde theory explains many pelvic lesions but does not fully account for extra-pelvic endometriosis. Alternative mechanisms include coelomic metaplasia, hematogenous and/or lymphatic spread, and embryonic cell remnants in the umbilical fold [1,2,4-6,8,9].

The differential diagnosis of umbilical endometriosis includes keloid, pyogenic granuloma, lipoma, seborrheic keratosis, lymphoma, urachal cyst, umbilical metastasis (Sister Mary Joseph nodule), and melanoma [2,3,7-9]. Dermoscopy has been reported to be a useful adjunct in the evaluation of umbilical lesions suspicious for endometriosis. Such dermoscopic findings have been described as red, homogeneous coloration that fades toward the periphery, with smaller, globular, darker structures termed “red atolls” within the background [3,7]. As cutaneous endometriosis can mimic melanoma or other pigmented lesions, dermoscopy may aid in distinguishing these entities. Surgical excision is the gold standard treatment and is associated with low recurrence rates and a reduced risk of malignant transformation [2-5,8-10]. Excision is typically performed via a transcutaneous periumbilical approach with free margins and should include the fascia and peritoneum [9,10]. Medical treatment with oral contraceptives or GnRH analogs may help reduce lesion size and can be used as a diagnostic tool itself [3,6,9].

The presence of painful lesions, cyclic bleeding, or concomitant pelvic pain should prompt consideration of cutaneous endometriosis, particularly in women of reproductive age. One systematic review found that the most common complaints of umbilical endometriosis were pain and catamenial symptoms, which were reported in approximately 83% of cases [4]. However, the clinical presentation can vary considerably, ranging from painful lesions that bleed to asymptomatic lesions with spontaneous, noncyclic bleeding, as illustrated by our patient. This variability can lead to misdiagnosis and delays in appropriate treatment. Because our patient had no prior surgical scar, secondary umbilical endometriosis was considered less likely, and Villar’s nodule was not included in the original differential diagnosis. Given the high rates of concomitant pelvic and extra-pelvic endometriosis, a diagnosis of umbilical endometriosis should prompt a gynecologic referral and workup, which typically includes pelvic examination and pelvic imaging with ultrasound or MRI [7,9,10]. The American College of Obstetricians and Gynecologists (ACOG) has recently updated its clinical practice guidelines on endometriosis, with an important shift to symptom-based clinical diagnosis over traditional reliance on surgery and tissue analysis [1]. One of the extra-pelvic symptoms included in the guidelines is cutaneous swelling or masses at prior surgical scars [1]. Clinicians encountering umbilical lesions should thus maintain a high index of suspicion for endometriosis, as early recognition may improve fertility and other health outcomes.

## Conclusions

Although cutaneous endometriosis is a rare and underrecognized entity, Villar’s nodule accounts for a substantial proportion of cutaneous cases and should be included in the differential diagnosis when evaluating umbilical lesions in women of reproductive age. While most cases of cutaneous endometriosis are linked to prior surgery, spontaneous formation cannot be excluded, as was the case in our patient. Careful history and physical examination, with biopsy and excision, are critical for early diagnosis and prevention of malignant transformation. Prompt diagnosis with appropriate gynecologic referral to assess concomitant pelvic endometriosis is essential, especially for women who desire future pregnancy.
